# Temperature-Dependent Pre-Bloodmeal Period and Temperature-Driven Asynchrony between Parasite Development and Mosquito Biting Rate Reduce Malaria Transmission Intensity

**DOI:** 10.1371/journal.pone.0055777

**Published:** 2013-01-31

**Authors:** Krijn P. Paaijmans, Lauren J. Cator, Matthew B. Thomas

**Affiliations:** Center for Infectious Disease Dynamics and Department of Entomology, Pennsylvania State University, University Park, Pennsylvania, United States of America; Institut Pasteur, France

## Abstract

A mosquito needs to bite at least twice for malaria transmission to occur: once to acquire parasites and, after these parasites complete their development in their mosquito host, once to transmit the parasites to the next vertebrate host. Here we investigate the relationship between temperature, parasite development, and biting frequency in a mosquito-rodent malaria model system. We show that the pre-bloodmeal period (the time lag between mosquito emergence and first bloodmeal) increases at lower temperatures. In addition, parasite development time and feeding exhibit different thermal sensitivities such that mosquitoes might not be ready to feed at the point at which the parasite is ready to be transmitted. Exploring these effects using a simple theoretical model of human malaria shows that delays in infection and transmission can reduce the vectorial capacity of malaria mosquitoes by 20 to over 60%, depending on temperature. These delays have important implications for disease epidemiology and control, and should be considered in future transmission models.

## Introduction

For malaria parasites to be transmitted from human to human requires at least two mosquito bites. First, to become infected the mosquito needs to feed on an infectious human host. The first bloodmeal that can potentially infect a mosquito is usually consumed 2 days after mosquito emergence from its aquatic breeding site [1]–[3], but this is likely to be temperature-dependent [4]. To then onwardly transmit, the mosquito needs to take another bloodmeal when it is infectious. Transmission to a new host cannot occur until the Extrinsic Incubation Period (EIP) of the parasite is completed. The EIP is the length of time it takes the malaria parasite to complete its development within the mosquito and migrate to the salivary glands, and is one of the key rate limiting steps in the transmission of malaria. EIP is known to be strongly temperature sensitive, taking anywhere from around 10 days to >30 days depending on conditions [5]–[7].

Human malaria mosquitoes (*Anopheles* spp.) generally exhibit discrete feeding cycles in which blood feeding only occurs at the beginning of a gonotrophic cycle and then not again until the blood has been digested, a batch of eggs has matured and oviposition is completed (e.g. [8]). While there is the possibility of a small proportion of *Anopheles* mosquitoes taking multiple blood feeds within a cycle [9], [10], evidence suggests this behavior might be restricted to smaller (and more energy depleted) mosquitoes during the first feeding cycle, immediately after emergence from their breeding site [11].

In most cases blood feeding is coupled to reproduction, and therefore the frequency of possible transmission events (i.e. acquiring the parasite in an early feeding event and then passing it on in a later feed) depends on the duration of this gonotrophic cycle. Again, the length of the gonotrophic cycle is strongly temperature dependent [7], [12]. Under warmer conditions (∼30°C), the gonotrophic cycle can be completed in just 2–3 days [12] resulting in a high frequency of blood feeding. Under cooler conditions (15–20°C), on the other hand, blood feeding might occur only once every 6–13 days [12], [13].

While both parasite development and the gonotrophic cycle are strongly affected by temperature, these processes are independent of one another and the nature of their temperature dependence differs (see results). There exists the possibility, therefore, that the EIP and feeding could be out of phase such that the parasite could complete development at an early or mid-point of a gonotrophic cycle. In this case, the parasite has a ‘waiting period’ where it cannot be transmitted until the next feed, even though the time since the initial infected bloodmeal has extended beyond the EIP.

Widely used malaria transmission models, such as the Ross-MacDonald models (see [14] for an overview of this family of models and [15]–[19] for illustrative applications), do not consider delays in infection due to a pre-bloodmeal period, or possible delays in onward transmission due to asynchrony between parasite development and the gonotrophic cycle. A limited number of models have included such delays, but have not explored the implications for transmission explicitly [20], [21]. The aim of the current study, therefore, is to address this knowledge gap.

We begin with some illustrative, proof-of-principle empirical investigations using the Asian malaria vector, *Anopheles stephensi*, and a rodent malaria, *Plasmodium yoelii*, to explore the assumptions that the pre-blood meal period is temperature-dependent, blood feeding is linked to the gonotrophic cycle, and that the EIP and the gonotrophic cycle are affected differentially by temperature. We then extend the study using some simple modifications of an established model for vectorial capacity. Using previously defined relationships between temperature and the gonotrophic cycle, and temperature and the EIP of the human malaria *P. falciparum*, we explore how biting and EIP can move in and out of phase across different thermal environments. This effect, combined with an initial temperature-dependent pre-bloodmeal period, can lead to substantial reductions in transmission intensity.

## Materials and Methods

### I. Empirical Studies on Pre-bloodmeal Period, Gonotrophic Cycle, Feeding Propensity and Extrinsic Incubation Period (EIP)

*Anopheles stephensi* mosquitoes were used throughout and were reared as described in Bell *et al*. [22].

#### Pre-bloodmeal Period

To test whether time to first blood meal is tied to temperature, we examined the daily feeding propensity of mosquitoes following emergence. *An. stephensi* pupae were placed at 18, 26 or 32±0.5°C in incubators (Percival Scientific Inc., USA), at 90±5% relative humidity and a 12L∶12D photoperiod. The following day, approx. 60 females and 30 males (<12*h* old) were placed in a cage (3 cages per temperature treatment), and kept at their respective temperatures, with glucose water provided *ad libitum*. Temperature was monitored closely with temperature loggers (OM-62, Omega, USA) at 15 min intervals.

Over the following 2–4 days the mosquitoes were presented daily with an opportunity to feed for 10 minutes (arm KPP). The number of mosquitoes that took a bloodmeal were scored, and those mosquitoes were discarded. In addition, the number of dead mosquitoes was quantified daily. Because it was not possible to offer the blood meal within the small reach-in chambers, the feeds were carried out in a walk-in climate chamber (Conviron, Canada), set to the appropriate baseline temperature for each treatment. Mosquitoes were allowed to adjust for 10 minutes before a bloodmeal was offered. The average time taken for the mosquitoes to take their first blood feed (the pre-bloodmeal period) was compared across temperatures using a Kruskal–Wallis test.

#### Gonotrophic Cycle

The length of the gonotrophic cycle (time between blood feeding and oviposition) was assessed at 3 temperatures (22, 24 and 26±0.5°C) that match previous empirical work on rodent malaria in our lab (see below). We studied the length of both the first and second cycle, as it has been reported that the first cycle is longer than the second and all subsequent gonotrophic cycles [13]. Experiments were carried out in incubators (Percival Scientific Inc., USA), at 90±5% relative humidity and a 12L∶12D photoperiod. Temperature was monitored closely with temperature loggers (OM-62, Omega, USA) at 15 min intervals.

Approximately 600 3–4 day old female *Anopheles stephensi* mosquitoes were allowed to blood feed (arm KPP) for ∼15 minutes at the beginning of the night cycle (6PM). After the feed, fully engorged mosquitoes were evenly distributed over four temperature treatments (∼130 mosquitoes per temperature). Fifty females were placed individually in plastic 5 mL tubes (diameter 1.5 cm, height 6 cm) to study the length of the first gonotrophic cycle. The tubes contained 1.5 mL of distilled water, a small filter-paper cone (height 2 cm) as an oviposition substrate, and were closed with a cotton wool ball. Tubes were monitored daily for eggs, and the length of the gonotrophic cycle (time between bloodmeal and first eggs) was recorded. The remaining females were kept in a cage, fed *ad libitum* on distilled water, and were provided with an oviposition medium.

As soon as the majority of the mosquitoes (>90%) in the tubes laid eggs, the mosquitoes in the cages were allowed to blood feed again for ∼15 min. After the feed, 50 females (per temperature treatment) were placed individually in tubes again to assess the length of the second gonotrophic cycle. Gonotrophic cycle lengths were compared across temperatures using a Mann-Whitney U test.

#### Feeding Propensity

To test whether blood feeding was tied to the gonotrophic cycle, or whether feeding could occur within a cycle, we examined the daily feeding propensity of mosquitoes following a bloodmeal. Three-to-four day-old female *An. stephensi* mosquitoes were blood fed and then distributed between cages (4 cages per temperature treatment, 30 mosquitoes per cage) maintained at 22, 24 or 26±0.5°C (walk-in climate chambers, Conviron, Canada), with 10% glucose water provided *ad libitum*. Relative humidity was 85±5%, the photoperiod 12L∶12D. Temperature was monitored closely with temperature loggers (OM-62, Omega, USA) at 15 min intervals.

Over the following 5–6 days the mosquitoes were presented daily with a feeding stimulus comprising a 250 ml flask filled with hot tap water (50–55°C) placed adjacent to a cage. This stimulus provides a heat cue and is a routine technique for attracting actively blood-seeking females in our laboratory. The number of mosquitoes recruiting to the heat source and observed actively probing through the mesh wall of the cage was recorded 2, 5 and 10 minutes after the feeding stimulus was presented, and the highest proportion responding (per day, per cage and per temperature) was used in the analysis [8].

The proportions of females attempting to feed on day 3 were compared using a Chi-Squared test. Analyses were performed with the statistical software IBM SPSS (v20).

#### Extrinsic Incubation Period

For estimates of EIP we utilized recently published data examining parasite development times of the rodent malaria, *P. yoelii* [18]. Full experimental methods are given elsewhere [18] but in brief, *An. stephensi* mosquitoes were fed on infectious mice and then maintained at 22, 24 or 26±1°C. Infected mosquitoes were dissected periodically from the different temperature treatments to determine the rate of oocyst maturation. At the point at which oocysts looked mature and ready to rupture, the salivary glands of infected mosquitoes were dissected to determine the presence of sporozoites (the completion of EIP). Salivary glands were dissected over at least 5 subsequent days to capture the cumulative sporozoite release. The proportion of females that harbored sporozoites in the salivary glands (prevalence) each day was used to create a distribution of EIP. For the purpose of the current paper, the EIP data were scaled to ‘the maximum prevalence of infectious mosquitoes’ recorded at a given temperature [18].

#### Ethical Issues

This study was carried out in accordance with the recommendations in the Guide for the Care and Use of Laboratory Animals of the NIH. The protocol was approved by the Animal Care and Use Committee of the Pennsylvania State University (Permit Number: 27452). The Pennsylvania State University Institutional Review Board determined that the experiments whereby mosquitoes were fed by arm do not meet the criteria for human subjects research and thus, do not require human subjects approval.

### II. Physiological Models of EIP, the Gonotrophic Cycle and Vectorial Capacity

Mosquito biting rate (daily feeding rate of a vector on a host, *a*) and the time from a vector becoming infected to becoming infectious (parasite extrinsic incubation period, *EIP*) are known to be strongly affected by temperature (*T*). We used established thermal performance curves available in the literature to describe these relationships. Biting rate, or its inverse *F* (feeding cycle length, in days), is described for *Anopheles pseudopunctipennis*, one of the main malaria vectors in South America [12], [19]:
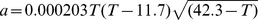
(1)The EIP of *P. falciparum* was calculated as the inverse of pathogen development rate (*PDR*, per day), as a function of temperature [19]:
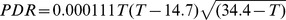
(2)


Mosquito biting rates and parasite development rates were calculated using mean temperatures ranging from 18–35°C, which covers the thermal limits for transmission of *P. falciparum* [19], [23], [24]. Parasite development rate equals zero at temperatures >34.4°C (see equation 2).

#### Delays in infection and transmission

Vectors typically require some time between emergence from their aquatic immature habitat and the uptake of their first bloodmeal [1]–[4]. The pre-bloodmeal period (or infection delay, *δ_i_*) is likely to be temperature-sensitive [4]. However, there are no published data describing this relationship for *Anopheles* mosquitoes and so we use our own empirical data presented in this paper (see results). Our proof-of-principle experiments were conducted across three temperatures only and we acknowledge that more research is clearly warranted to better define this relationship (see discussion). Nonetheless the data clearly show that the relationship is nonlinear (see reference [4] also, for data on *Aedes* mosquitoes). Therefore, we fitted a nonlinear polynomial regression through our empirical data:
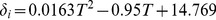
(3)As we know the temperature-sensitivity of both EIP and *a* (equations 1 and 2), we can estimate the transmission delay (*δ_t_*), or waiting period (time between a pathogen finishing its development within the mosquito, and the next mosquito bite), with the following ‘ceiling’ function:
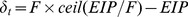
(4)As an example, if EIP is 16 days, and *F* 5 days, the EIP is completed at 3.2 (16/5) gonotrophic cycles. By rounding this value up ‘ceil(*EIP*/*F*)’, we get that transmission occurs at the end of the 4^th^ cycle. The transmission delay is then 5×4–16 = 4 days.

Adding *δ_t_* to *EIP* gives total time from infection to actual transmission of a parasite by a mosquito.

#### Vectorial Capacity

How effectively mosquito populations transmit malaria can be quantified by the vectorial capacity (*C*) equation:
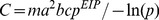
(5)where *m* is the mosquito∶human ratio, *a* mosquito biting frequency, *bc* mosquito vector competence (*b* describes the probability of a person becoming infected via a bite from an infectious vector, and *c*, the probability of a vector becoming infected by feeding on an infectious person), *p* daily mosquito survival rate, and EIP parasite development time or extrinsic incubation period inside the mosquito host [6]. In the absence of (human) host recovery, *C* represents the number of new infectious mosquito bites that arise from one infected person introduced into a population of susceptible hosts.

The delays in infection and transmission will impact on the number of mosquitoes still alive to transmit once the EIP is completed (*p^EIP^* in equation 5). We can link mosquito biting with pathogen infection/transmission opportunities by adding the delays to equation 5, arriving at the following:
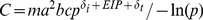
(6)


This initial model follows the standard approach of defining EIP as a single value for a given temperature. However, parasite invasion of the mosquito salivary glands follows a cumulative distribution [18] and it is not entirely clear at what point parasites can be successfully transmitted along this distribution. Accordingly, we present as a limiting case another scenario whereby parasites have to wait a full feeding cycle after completing their development before being transmitted (this approach defines the maximum potential waiting time of a cohort of parasites):
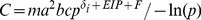
(7)


We modeled the relative effects of our different models (equations 5, 6 and 7) on vectorial capacity, using the temperature-sensitive *R_0_* model that has recently been developed by Mordecai and colleagues [19].

All model calculations and figures were produced in the statistical package *R* [25].

## Results

### Empirical Studies on Pre-Bloodmeal Period, Gonotrophic Cycle Length, Feeding Propensity and EIP

**Figure 1:**
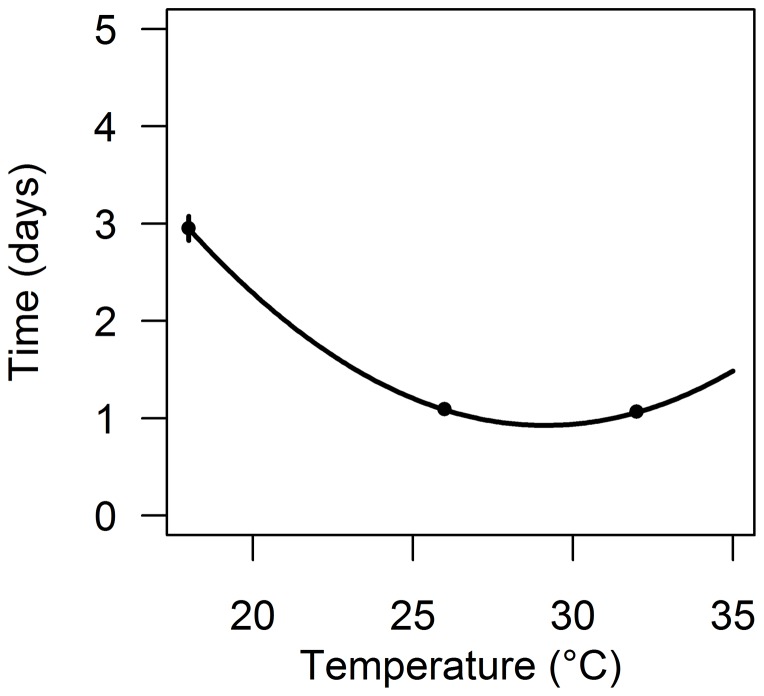
Temperature-dependency of the pre-bloodmeal period. The mean time between mosquito emergence and first bloodmeal (infection delay or *δ_i_*) at 18, 26 and 32°C. The black line represents a polynomial regression (see methods).

The pre-bloodmeal period (i.e. the time between mosquito emergence and first bloodmeal) differed across temperature (Kruskal–Wallis test, *P* = 0.027). The infection delay can potentially be 2 days longer at 18°C, compared to 26°C and 32°C (Figure 1).

**Figure 2:**
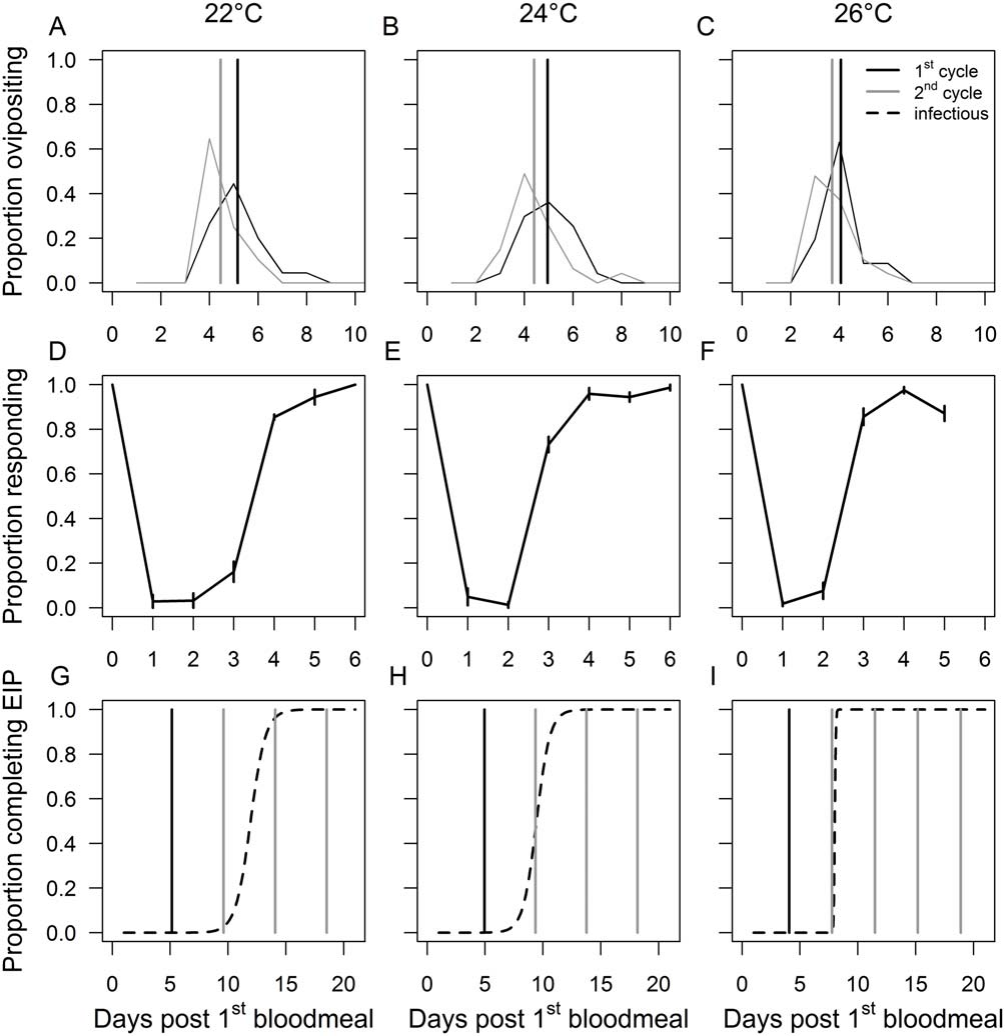
Gonotrophic cycle length, feeding propensity and EIP across temperature. A–C: Length of the first and second gonotrophic (or feeding) cycle of *Anopheles stephensi*, D–F: Proportion of blood fed *An. stephensi* responding to feeding stimulus (on days 1–6), G–I: proportion of infectious mosquitoes over time, overlaid with mean length of the first (black line) and subsequent cycles (grey lines), derived from A–C, at 22, 24 and 26°C.

The duration of the first gonotrophic cycle was longer than that of the second across all temperature treatments (Mann Whitney U test, *P*<0.05). The average length of the first gonotrophic cycle was 5.2, 5.0 and 4.1 days at 22, 24 and 26°C, respectively, while the second cycle (and we assume all further cycles) was 4.5, 4.4 and 3.7 days, respectively (Figure 2A–C).

Female mosquitoes showed a marked crash in propensity to feed 1–2 days after a blood meal followed by a gradual recovery over the next 3–5 days (Figure 2D–F). The rate of recovery was faster as temperature increased. For example, on day 3 post bloodmeal, only 14.75±11.4% of females responded in the 22°C treatment, compared to 73.25±6.9% at 24°C and 85.55±7.3% at 26°C (χ^2^ = 93.2, d.f. = 2, *P*<0.001). These results indicate that feeding intervals are strongly linked to the duration of the gonotrophic cycle.

The EIP data show that the time taken for 90% of mosquitoes to become infectious after an infected blood meal varies with temperature, taking 13, 11 and 8 days at 22, 24 and 26°C, respectively (Figure 2G–I) [18]. Overlaying the feeding cycle data with these EIP data reveals three different patterns across temperature. At 22°C, the parasite EIP synchronizes well with the feeding cycle, such that mosquitoes are predicted to be ready to feed at the point where the maximum proportion of mosquitoes become infectious. At 26°C, on the other hand, EIP is completed just after a feeding cycle resulting in a ‘waiting period’ for the parasite until the next feed, even though sporozoites are in the salivary glands. At 24°C, an intermediate pattern occurs whereby the feeding cycle falls at the mid-point of the cumulative EIP curve.

### Temperature-Driven Delays in Infection and Transmission of Human Malaria and Consequences for Vectorial Capacity

**Figure 3:**
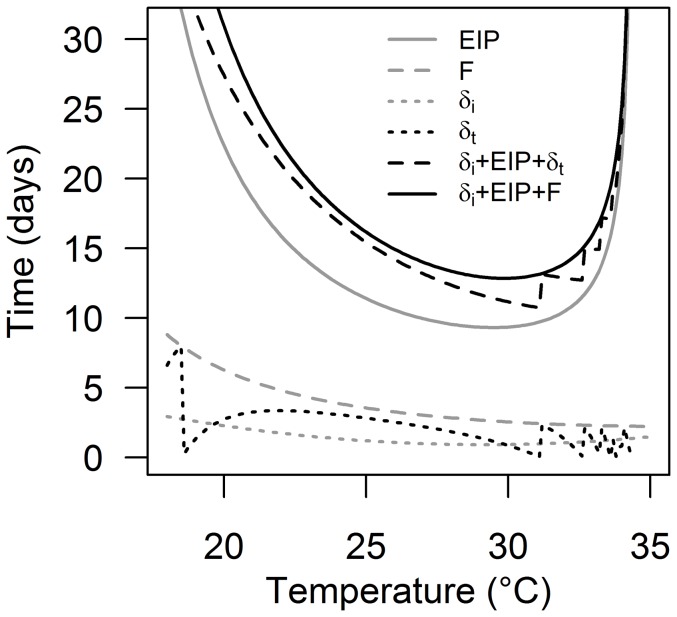
Temperature-driven asynchrony between malaria parasite development and mosquito biting rate. The time between mosquito emergence and first bloodmeal (infection delay or *δ_i_*), the length of the extrinsic incubation period of the parasite (EIP), time interval between mosquito feeding events (F), the time between completion of EIP and the next mosquito bite (transmission delay or *δ_t_*), time between mosquito emergence and parasite transmission occurring (*δ_i_*+EIP+*δ_t_*), and the maximum potential waiting time between mosquito emergence and parasite transmission (parasites have to wait for a full feeding cycle before being transmitted; *δ_i_*+EIP+F).

The empirical data demonstrate the potential for temperature to (1) affect the pre-bloodmeal period, and (2) create asynchrony between completion of EIP and a potential transmission event (i.e. blood feeding). In Figure 3 we use established temperature-dependent relationships for EIP and feeding rate, *F* [19], to explore the extent of the possible transmission delays resulting from this asynchrony for the human malaria parasite, *P. falciparum*. This figure reveals that the delay (*δ_t_*) could range from 0 (biting and parasite development are perfectly in sync) to 8 days (the length of a full feeding cycle), depending on temperature.

**Figure 4:**
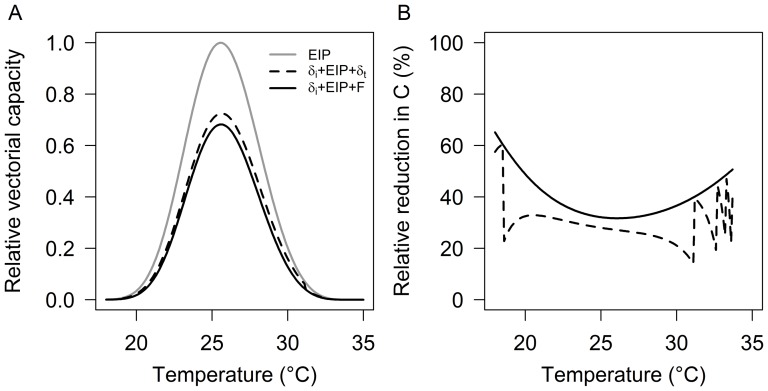
Effect of infection and transmission delays on malaria transmission intensity. (A) Relative vectorial capacity of malaria mosquitoes using (*i*) the conventional approach (EIP only; equation 5), (*ii*) including infection and transmission delays (*δ_i_*+EIP+*δ_t_*; eq. 6), and (*iii*) including a ‘maximal’ wait (*δ_i_*+EIP+F; eq. 7). (B) Relative reductions in vectorial capacity compared to the conventional approach.

Including the temperature-driven delays in infection and transmission dramatically lowers the estimates of vectorial capacity relative to conventional models that assume no delays (Figure 4A). Depending on temperature, reductions in vectorial capacity range from approximately 20 to 60% (Figure 4B). These reductions are larger when adult vector survivorship is lower; i.e. a shorter life expectancy means any delays in infection and transmission are more important (which includes mosquitoes potentially becoming infected during their second or third feed, rather than the first feed (data not shown)). The patterns of the reduction in vectorial capacity show marked discontinuities as EIP and feeding move in and out of phase across temperature. Accordingly, even subtle shifts in temperature can result in very large variations in transmission intensity, especially towards the temperature extremes.

## Discussion

Our illustrative empirical data and simple modeling analysis suggest the potential for temperature to impact pre-bloodmeal period, and hence time of infection. In addition temperature can have differential impacts on mosquito feeding and parasite development (EIP), leading to variable delays in parasite transmission following the initial infectious blood meal. Other studies on vector-borne diseases have considered delays for initial infection of the vector [3], [26] and/or subsequent transmission [21], [26], [27] due to variation in timing and frequency of feeding. The implications of such delays for malaria transmission intensity (vectorial capacity or basic reproductive number) have not previously been highlighted.

Including a pre-feed delay reduces vectorial capacity across the board, as it results in fewer mosquitoes living long enough to be able to transmit the parasite. The exact patterns will depend critically on the specifics of mosquito biology yet for many traits there is a lack of good empirical data. For example, our studies show that pre-infection delay (*δ_i_*) can range from 1 to 3 days, depending on temperature. However, in a study on *Aedes albopictus*, a known mosquito vector of dengue virus and chikungunya, the average pre-bloodmeal period ranged from 15 days at 15°C to 4.2 days at 30°C, and then increased again at even higher temperatures [4]. These latter data were derived from life table studies whereby immature stages were reared at the different temperatures. These immatures might therefore carry over resources to the adult stage magnifying the adult stage-specific effects we report. If a similar pattern occurs for malaria mosquitoes, the relative importance of *δ_i_* will increase, especially toward the temperature extremes.

Adding the differential effects of temperature on parasite development and feeding leads to further reductions. At the extreme, where transmission is always delayed by a full feeding cycle, vector competence is reduced by ∼30–60%, depending on the temperature. If the delays depend more specifically on the synchrony between EIP and feeding, the effects on vectorial capacity are much more variable. The addition of ‘waiting periods’ is never predicted to increase transmission relative to conventional models without delays, but the discrete nature of feeding episodes means that vectorial capacity can exhibit dramatic shifts as feeding and EIP move in and out of phase. We acknowledge that both gonotrophic cycle and EIP actually follow distributions around their means, which could shape these patterns, but we still expect asynchrony to add considerable variability as proportions of mosquito cohorts miss the transmission window.

We also used a single temperature-dependent model for the first gonotrophic cycle that derives from studies on one species of malaria vector [12], [19]. Good empirical data on the relationship between temperature and the length of the second and subsequent cycles are lacking but would clearly be useful for improving understanding of transmission. In addition, it is unclear whether there is variation in the relative thermal sensitivity or the absolute duration of the gonotrophic cycles between malaria mosquito species. The same applies for the EIP, which is assumed to be a fixed property of the parasite species. However, there is evidence for adaptation between malaria clones and local vectors [28], [29], which could result in spatial and/or temporal deviations in the temperature-EIP relationship. More broadly, factors such as heterogeneous biting behavior of mosquitoes [30], age-dependent mosquito mortality [3], feeding-dependent mortality [31], degree of anthropophily [32] and even possible parasite manipulation of blood feeding behavior [33], all have the potential to alter vectorial capacity via effects on the feeding-EIP relationship. Exploring this full suite of effects would benefit from application of more biologically realistic stage-structured or feeding cycle models (e.g. [34], [35]), rather than the simple vectorial capacity approach used here.

These limitations notwithstanding, studies in western Kenya indicate that asynchrony between mosquito feeding and parasite development can occur in the field (see [13], [17]). Given the potential significance for measures of transmission intensity and subsequent consequences for malaria control and possible elimination strategies, future modeling and empirical studies should seek to better understand the phenomena presented in this paper. Such effects have potentially important implications for disease epidemiology since even minor changes in temperature could lead to large variation in dynamics over time or space, potentially making overall trends (e.g. from climate warming) difficult to interpret.

## References

[1] Stone CM, Hamilton IM, Foster WA (2011) A survival and reproduction trade-off is resolved in accordance with resource availability by virgin female mosquitoes. Animal Behaviour 81: 765–774. 21499504 10.1016/j.anbehav.2011.01.008 PMC3074587

[2] Fernandes L, Briegel H (2005) Reproductive physiology of *Anopheles gambiae* and *Anopheles atroparvus* . Journal of Vector Ecology 30: 11–26. 16007951

[3] Bellan SE (2010) The importance of age dependent mortality and the extrinsic incubation period in models of mosquito-borne disease transmission and control. PLoS ONE 5: e10165. 20405010 10.1371/journal.pone.0010165 PMC2854142

[4] Delatte H, Gimonneau G, Triboire A, Fontenille D (2009) Influence of temperature on immature development, survival, longevity, fecundity, and gonotrophic cycles of *Aedes albopictus*, vector of chikungunya and dengue in the Indian Ocean. Journal of Medical Entomology 46: 33–41. 19198515 10.1603/033.046.0105

[5] Boyd MF (1949) Epidemiology: factors related to the definitive host. In: Boyd MF, editor. Malariology: A comprehensive survey of all aspects of this group of diseases from a global standpoint. Philadelphia: W.B. Saunders Company. pp. 608–697.

[6] MacDonald G (1957) The epidemiology and control of malaria. London, UK: Oxford University Press. 201 p.

[7] Detinova TS (1962) Age-grouping methods in diptera of medical importance. Geneva: World Health Organization. 216 p. 13885800

[8] Blanford S, Shi W, Christian R, Marden JH, Koekemoer LL, et al (2011) Lethal and pre-lethal effects of a fungal biopesticide contribute to substantial and rapid control of malaria vectors. PLoS ONE 6: e23591. 21897846 10.1371/journal.pone.0023591 PMC3163643

[9] Scott TW, Githeko AK, Fleisher A, Harrington LC, Yan G (2006) DNA profiling of human blood in anophelines from lowland and highland sites in western Kenya. The American Journal of Tropical Medicine and Hygiene 75: 231–237. 16896124

[10] Norris LC, Fornadel CM, Hung W-C, Pineda FJ, Norris DE (2010) Frequency of multiple blood meals taken in a single gonotrophic cycle by *Anopheles arabiensis* mosquitoes in Macha, Zambia. The American Journal of Tropical Medicine and Hygiene 83: 33–37. 20595474 10.4269/ajtmh.2010.09-0296 PMC2912572

[11] Takken W, Klowden MJ, Chambers GM (1998) Effect of body size on host seeking and blood meal utilization in *Anopheles gambiae* sensu stricto (Diptera: Culicidae): the disadvantage of being small. Journal of Medical Entomology 35: 639–645. 9775585 10.1093/jmedent/35.5.639

[12] Lardeux FJ, Tejerina RH, Quispe V, Chavez TK (2008) A physiological time analysis of the duration of the gonotrophic cycle of *Anopheles pseudopunctipennis* and its implications for malaria transmission in Bolivia. Malaria Journal 7: 141. 18655724 10.1186/1475-2875-7-141 PMC2518372

[13] Afrane YA, Lawson BW, Githeko AK, Yan G (2005) Effects of microclimatic changes caused by land use and land cover on duration of gonotrophic cycles of *Anopheles gambiae* (Diptera: Culicidae) in western Kenya highlands. Journal of Medical Entomology 42: 974–980. 16465737 10.1093/jmedent/42.6.974

[14] Smith DL, Battle KE, Hay SI, Barker CM, Scott TW, et al (2012) Ross, Macdonald, and a theory for the dynamics and control of mosquito-transmitted pathogens. PLoS Pathogens 8: e1002588. 22496640 10.1371/journal.ppat.1002588 PMC3320609

[15] Martens P, Kovats RS, Nijhof S, de Vries P, Livermore MTJ, et al (1999) Climate change and future populations at risk of malaria. Global Environmental Change 9: S89–S107.

[16] Parham PE, Michael E (2010) Modeling the effects of weather and climate change on malaria transmission. Environmental Health Perspectives 118: 620–626. 20435552 10.1289/ehp.0901256 PMC2866676

[17] Afrane YA, Little TJ, Lawson BW, Githeko AK, Yan GY (2008) Deforestation and vectorial capacity of *Anopheles gambiae* giles mosquitoes in malaria transmission, Kenya. Emerging Infectious Diseases 14: 1533–1538. 18826815 10.3201/eid1410.070781 PMC2573462

[18] Paaijmans KP, Blanford S, Chan BHK, Thomas MB (2012) Warmer temperatures reduce the vectorial capacity of malaria mosquitoes. Biology Letters 8: 465–568. 22188673 10.1098/rsbl.2011.1075 PMC3367745

[19] Mordecai E, Paaijmans K, Johnson L, Balzer C, Ben-Horin T, et al (2013) Optimal temperature for malaria transmission is dramatically lower than previously predicted. Ecology Letters 16: 22–30. 23050931 10.1111/ele.12015

[20] Killeen GF, McKenzie FE, Foy BD, Schieffelin C, Billingsley PF, et al (2000) A simplified model for predicting malaria entomologic inoculation rates based on entomologic and parasitologic parameters relevant to control. American Journal of Tropical Medicine and Hygiene 62: 535–544. 11289661 10.4269/ajtmh.2000.62.535 PMC2483339

[21] Hoshen MB, Morse AP (2004) A weather-driven model of malaria transmission. Malaria Journal 3: 32. 15350206 10.1186/1475-2875-3-32 PMC520827

[22] Bell AS, Blanford S, Jenkins N, Thomas MB, Read AF (2009) Real-time quantitative PCR for analysis of candidate fungal biopesticides against malaria: Technique validation and first applications. Journal of Invertebrate Pathology 100: 160–168. 19320043 10.1016/j.jip.2009.01.006 PMC2666797

[23] Guerra CA, Gikandi PW, Tatem AJ, Noor AM, Smith DL, et al (2008) The limits and intensity of *Plasmodium falciparum* transmission: Implications for malaria control and elimination worldwide. Plos Medicine 5: 300–311. 10.1371/journal.pmed.0050038 PMC2253602 18303939

[24] Paaijmans KP, Read AF, Thomas MB (2009) Understanding the link between malaria risk and climate. Proceedings of the National Academy of Sciences 106: 13844–13849. 10.1073/pnas.0903423106 PMC2720408 19666598

[25] R Development Core Team (2010) R: A language and environment for statistical computing. http://www.R-project.org/. Vienna, Austria: R Foundation for Statistical Computing.

[26] Napp S, Gubbins S, Calistri P, Allepuz A, Alba A, et al (2011) Quantitative assessment of the probability of bluetongue virus overwintering by horizontal transmission: application to Germany. Veterinary Research 42: 4. 21314966 10.1186/1297-9716-42-4 PMC3031226

[27] Ermert V, Fink A, Jones A, Morse A (2011) Development of a new version of the Liverpool Malaria Model. I. Refining the parameter settings and mathematical formulation of basic processes based on a literature review. Malaria Journal 10: 35. 21314922 10.1186/1475-2875-10-35 PMC3055220

[28] Joy DA, Gonzalez-Ceron L, Carlton JM, Gueye A, Fay M, et al (2008) Local adaptation and vector-mediated population structure in *Plasmodium vivax* malaria. Molecular Biology and Evolution 25: 1245–1252. 18385220 10.1093/molbev/msn073 PMC2386084

[29] Harris C, Morlais I, Churcher TS, Awono-Ambene P, Gouagna LC, et al (2012) *Plasmodium falciparum* produce lower infection intensities in local *versus* foreign *Anopheles gambiae* populations. PLoS ONE 7: e30849. 22292059 10.1371/journal.pone.0030849 PMC3266902

[30] Smith DL, McKenzie FE, Snow RW, Hay SI (2007) Revisiting the basic reproductive number for malaria and its implications for malaria control. Plos Biology 5: 531–542. 10.1371/journal.pbio.0050042 PMC1802755 17311470

[31] Dawes E, Churcher T, Zhuang S, Sinden R, Basanez M-G (2009) *Anopheles* mortality is both age- and *Plasmodium*-density dependent: implications for malaria transmission. Malaria Journal 8: 228. 19822012 10.1186/1475-2875-8-228 PMC2770541

[32] Kiware SS, Chitnis N, Moore SJ, Devine GJ, Majambere S, et al (2012) Simplified models of vector control impact upon malaria transmission by zoophagic mosquitoes. PLoS ONE 7: e37661. 22701527 10.1371/journal.pone.0037661 PMC3365128

[33] Cator LJ, Lynch PA, Read AF, Thomas MB (2012) Do malaria parasites manipulate mosquitoes? Trends in Parasitology 28: 466–470. 23044288 10.1016/j.pt.2012.08.004 PMC3478439

[34] Hancock PA, Thomas MB, Godfray HCJ (2009) An age-structured model to evaluate the potential of novel malaria-control interventions: a case study of fungal biopesticide sprays. Proceedings of the Royal Society B: Biological Sciences 276: 71–80. 18765347 10.1098/rspb.2008.0689 PMC2614244

[35] Eckhoff P (2011) A malaria transmission-directed model of mosquito life cycle and ecology. Malaria Journal 10: 303. 21999664 10.1186/1475-2875-10-303 PMC3224385

